# Chopper-Stabilized Instrumentation Amplifier with Automatic Frequency Tuning Loop

**DOI:** 10.3390/mi9060289

**Published:** 2018-06-08

**Authors:** Chen-Mao Wu, Hsiao-Chin Chen, Ming-Yu Yen, San-Ching Yang

**Affiliations:** 1 Asmedia Technology Inc., New Taipei 231, Taiwan; alexwuee2b@gmail.com; 2 Department of Electrical Engineering, National Taiwan University of Science and Technology, Taipei 10607, Taiwan; hcchen@mail.ntust.edu.tw; 3 Inventec Corporation, Taipei 11107, Taiwan; m10207414@mail.ntust.edu.tw

**Keywords:** automatic frequency tuning loop, chopper technique, instrumentation amplifier, low-pass filter

## Abstract

A variable-gain chopper-stabilized instrumentation amplifier (chopper IA), which employs a low pass filter (LPF) to attenuate the up-converted noise at the chopping frequency, is presented. The circuit is designed and fabricated with Taiwan Semiconductor Manufacturing Company (TSMC) (Hsinchu, Taiwan) 0.18 μm complementary metal-oxide-semiconductor (CMOS) technology. Consuming 1.1 mW from a 1.2 V supply voltage, the chopper IA achieves a variable gain of 20.7–48.5 dB, with a minimum bandwidth of 6.7 kHz and a common-mode rejection ratio (CMRR) of 95 dB below 10 kHz. By using the chopper technique, the input-referred noise of the chopper IA can be reduced to 0.28 μVrms (0~96 kHz), with a chopping frequency of 83.3 kHz. An automatic frequency tuning loop (ATL) is employed to adjust the corner frequency of the LPF dynamically so that the frequency ratio between the chopping frequency and the LPF corner frequency is 8.3, ensuring a noise reduction of 36.7 dB.

## 1. Introduction

To meet a variety of healthcare demands, physiological signal acquisition can be performed by implantable, wearable, or portable monitoring systems [1,2]. Recently, systems that can monitor physiological signals, e.g., electroencephalography (EEG), electrocardiography (ECG), blood pressure and glucose, have been proposed and demonstrated [3,4,5]. Chopper IAs are widely adopted in these systems for their advantageous low noise, high input impedance, and high common mode rejection ratio (CMRR).

The chopper modulation operation is describeted as follows [6]: The bandwidth of the signal is assumed to be less than half the chopping frequency. The input chopper up-converts the signal to the chopping frequency and its odd harmonics, and then delivers it to the amplifier. After amplification, the signal is then down-converted to its original band by the output chopper. Because the DC offset and noise only go through the chopper modulation once, they are up-converted to the chopping frequency and its odd harmonics. The low-pass filter (LPF) then attenuates the up-converted noise. The amount of noise reduction depends on the ratio of the LPF corner frequency to the chopping frequency. However, both the corner frequency of the LPF and the chopping frequency may change due to process variations, which can diminish the amount of noise reduction.

In this work, strong correlation is built up between the corner frequency of the LPF and the chopping frequency so that the noise reduction is not affected by process variations. The rest of the paper is organized as follows. The system architecture and the way that the chopper IA cooperates with the automatic frequency tuning loop (ATL) to achieve the above-mentioned goal is introduced in Section 2. The design of the building blocks is addressed in Section 3. The measurement results are reported in Section 4. Finally, this work is summarized in Section 5.

## 2. System Architecture

**Figure 1:**
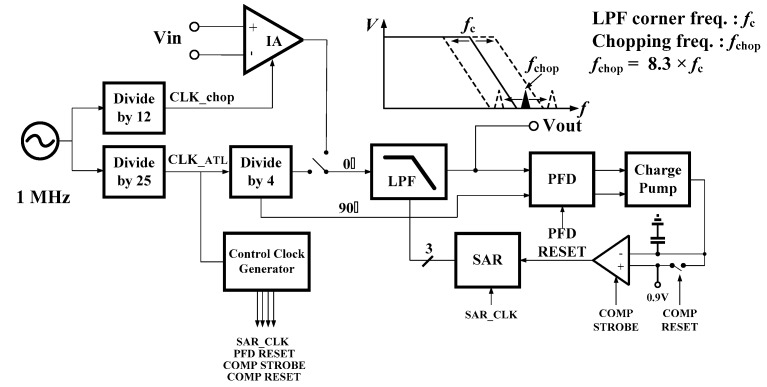
Block diagram of the proposed chopper IA with automatic frequency tuning loop (ATL).

A block diagram of the proposed chopper IA, which employs an ATL to control the corner frequency of the LPF for noise-reduction, is shown in Figure 1. CLK_chop is the chopping signal and CLK_ATL is the reference clock signal of the ATL. The former is generated from the configuration of an on-chip ring oscillator followed by a divide-by-12 circuit, while the latter is generated from the configuration of the same ring oscillator followed by a divide-by-25 circuit. The chopping frequency is represented by f_chop, and the frequency of the ATL reference clock, by f_ATL. Note that the corner frequency of the LPF is equal to one fourth of f_ATL. In this way, the ratio between the chopping frequency f_chop and the LPF corner frequency is always 8.3, even though the oscillating frequency of the ring oscillator changes with process variations. As a result, the LPF ensures an attenuation of 36.7 dB at the chopping frequency to provide adequate noise reduction in the chopper IA. In this work, an on-chip ring oscillator at 1 MHz is implemented. Therefore, the chopping signal of 83.33 kHz is delivered to the chopper IA, while the reference clock of 40 kHz is delivered to the ATL. During the operation, the input of the LPF is first connected to the divide-by-four circuit to form the frequency tuning loop for corner frequency calibration. After the calibration is completed, the input of the LPF is connected to the chopper IA for noise reduction.

In addition to the LPF, the ATL consists of a divide-by-four circuit, a phase frequency detector (PFD), a charge pump (CP), a comparator and a successive approximation register (SAR) [7,8]. The divide-by-four circuit generates a pair of quadrature signals at 10 kHz, namely, signals with 90° phase difference. In Figure 1, the one that is sent to the LPF is defined as the 0° signal and the other is defined as the 90° signal. When the 0° signal passes though the LPF, the filter introduces a frequency-dependent phase delay. Theoretically, the introduced phase delay should be 90° at the corner frequency of the LPF. Therefore, the frequency tuning/calibration can be performed by measuring the introduced phase delay. There are three key steps in each tuning cycle. First, the output signal of the LPF, the 0° signal with introduced phase delay, is compared with the 90° signal in the PFD. Secondly, the PFD output signals modulate the charge/discharge currents of CP, and the CP output is compared with a reference voltage of 0.9 V in the comparator to deliver a digital output. Then, the SAR logic sets one bit of the 3-bit control code of the LPF in accordance with this digital output signal. It takes three tuning cycles to complete the frequency tuning. Ideally, the Sallen-Key LPF would exhibit the corner frequency of 10 kHz after the frequency tuning.

## 3. Circuit Design

### 3.1. Chopper Instrumentation Amplifier: Chopper IA

**Figure 2:**
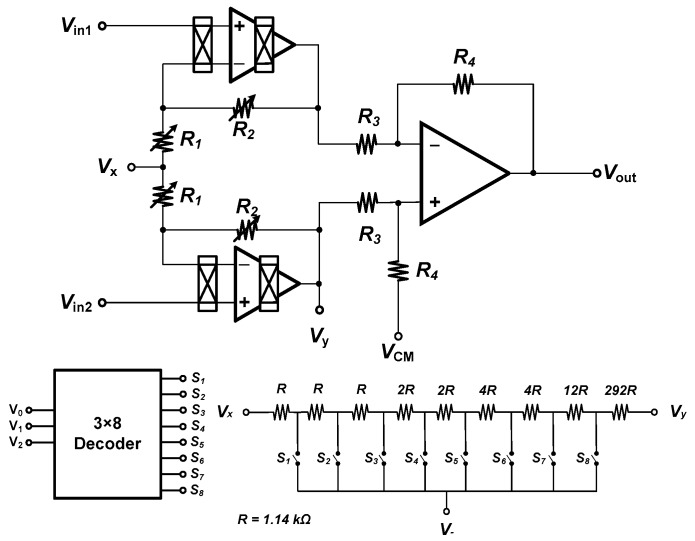
Tunable gain chopper IA [9].

The two-stage variable-gain chopper IA is developed from a difference amplifier, as depicted in Figure 2 [9]. The variable-gain function is realized by a switch-resistor array and a 3-to-8 decoder, where a unit resistor of 1.14 kΩ is adopted and the maximum resistance is 333 kΩ. The chopper stabilization technique is performed by using two chopper-stabilized operational amplifiers, OP1 and OP2, in the first stage because the noise of the first stage is most critical [9].

#### 3.1.1. Chopper Operational Amplifier

**Figure 3:**
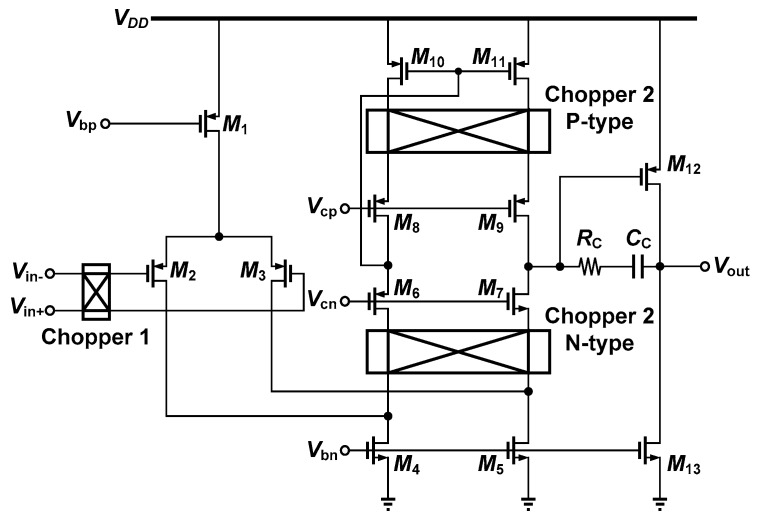
Chopper operational amplifier [9].

A schematic of the chopper-stabilized operational amplifier is shown in Figure 3 [9]. As previously mentioned, the noise performance of a two-stage amplifier is dominated in the first stage. If the noise origin of the operational amplifiers in the first stage can be removed, the noise performance of the IA can be significantly improved. The chopper modulator at the input, represented by chopper1, translates the input signal from its original band to the chopping frequency. The other two chopper modulators, chopper2 P-type and chopper2 N-type, then translate the desired signal back to its original band while converting the flicker noise, DC offsets, or noises well below the chopping frequency to the chopping frequency.

#### 3.1.2. Chopper Modulator

**Figure 4:**
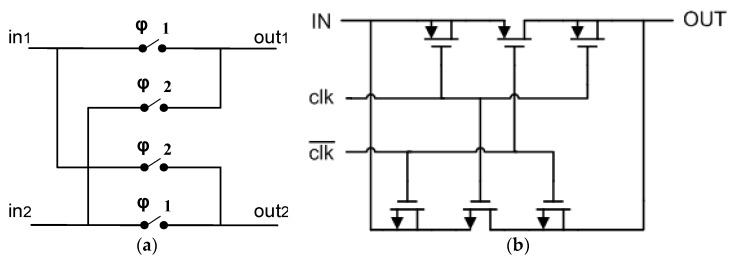
(**a**) Chopper modulation topology; (**b**) Switch topology.

The configuration of the chopper modulator and the switch implementation are shown in Figure 4a,b, respectively. To deal with imperfections such as the charge injection and clock feedthrough, dummies are added to the switches at both the sources and drains. When the switches are off, the channel charges are canceled by the dummies to prevent an electric potential error at the output. The switches are driven by non-overlapping clocks φ1 and φ2. During the operation, the input signals at nodes in1 and in2 are alternatively directed to either the node out1 or out2.

**Figure 5:**
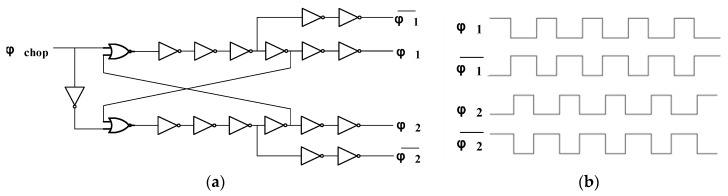
(**a**) Non-overlapping clock generator; (**b**) Non-overlapping clock signals.

The non-overlapping clock generator is shown in Figure 5a, and the waveform of the non-overlapping clock signals φ1 and φ2 is shown in Figure 5b. The non-overlapping clock generator is used to provide the required clocks φ1 and φ2 for the chopper modulator. The signal φ_chop_ is a square wave generated from the configuration of the on-chip ring oscillator followed by a divide-by-12 circuit, as previously mentioned. Notably, φ1 and φ2 need to be a pair of non-overlapping clock signals so that the switches driven by different clock signals are not turned on simultaneously. The inverting clock signals are also generated to control the dummy switches. The multi-inverter buffer stage is adopted to boost the driving capability of the clock signals.

**Figure 6:**
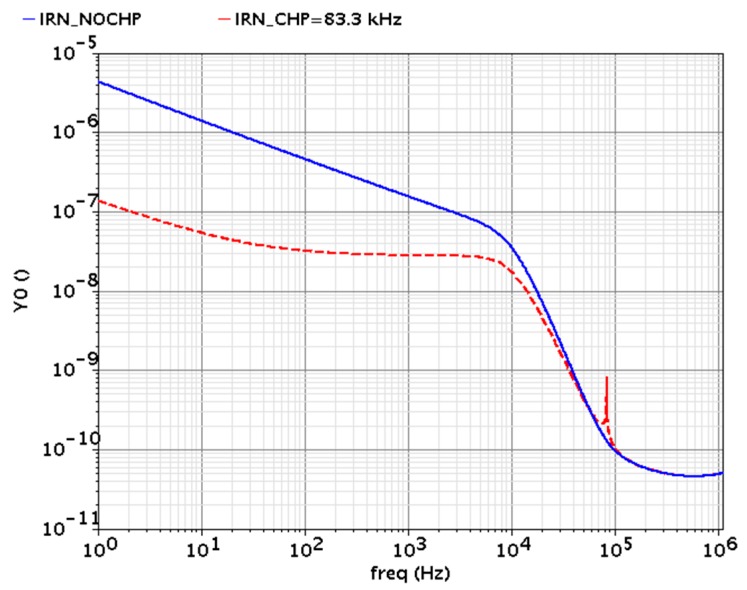
Input-referred noises of the chopper IA before (red dot line) and after (blue solid line) the chopper modulator is activated.

**Figure 7:**
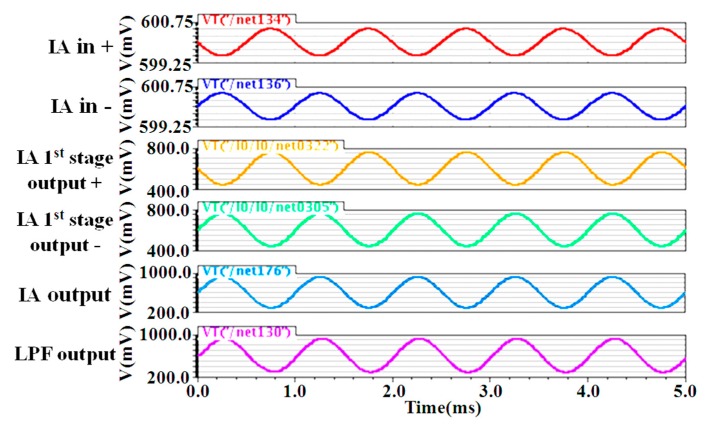
Transient simulation of the chopper IA.

The input-referred noise simulation results are obtained from the chopper IA before and after the chopper modulator are activated, as shown in Figure 6. The input-referred noise is greatly reduced after the chopper modulator is activated, with the chopping clock at 83.3 kHz. When the chopper modulator is activated, the input-referred noise at 100 Hz and 1 kHz are 32.2 nV/√Hz and 27.9 nV/√Hz, respectively. The transient simulation is also performed, as shown in Figure 7. A 1 kHz differential signal, with a DC level of 600 mV and an amplitude of 0.5 mV, is applied at the input of the chopper IA. According to the simulation results, the output signals exhibit ignorable distortion, where the total harmonic distortions observed at the IA output node and LPF output node are 74.6 dB and 51.6 dB, respectively.

### 3.2. Sallen-Key Low Pass Filter

**Figure 8:**
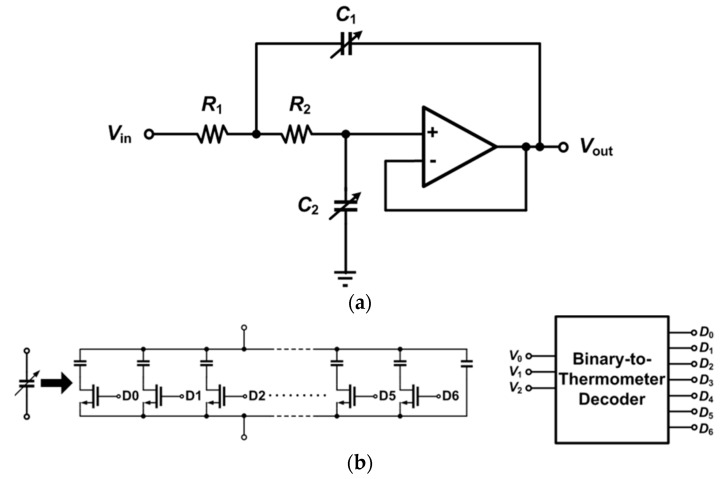
(**a**) Sallen-Key low-pass filter; (**b**) Cap-array schematic.

**Figure 9:**
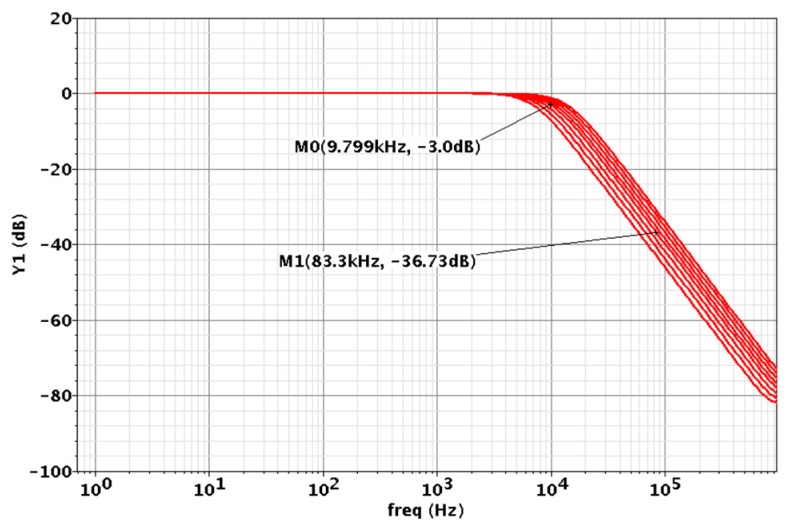
Frequency response simulation results of the low pass filter (LPF).

As the last stage in the chopper IA, the LPF attenuates the up-converted noise at the chopping frequency. A schematic of the Sallen-Key LPF [10], where both variable capacitors C1 and C2 are realized by 3-bit cap-arrays and R1–2 = 1 MΩ, is shown in Figure 8a. The schematic of the cap-array is shown in Figure 8b, where the capacitance of a unit capacitor is 104.0 fF. With a 3-bit binary-to-thermometer decoder, the LPF can be operated in 8 modes, where the capacitors C1 and C2 can be varied from 14.6 pF to 29.7 pF and 7.3 pF to 14.9 pF, respectively. The frequency response simulation results of the LPF are shown in Figure 9, where 8 different corner frequencies are realized. According to the simulation results, the corner frequency of the LPF can be varied from 6.9 kHz to 13.6 kHz with a step size of 0.8~1 kHz.

### 3.3. Divide-by-Four Circuit

**Figure 10:**
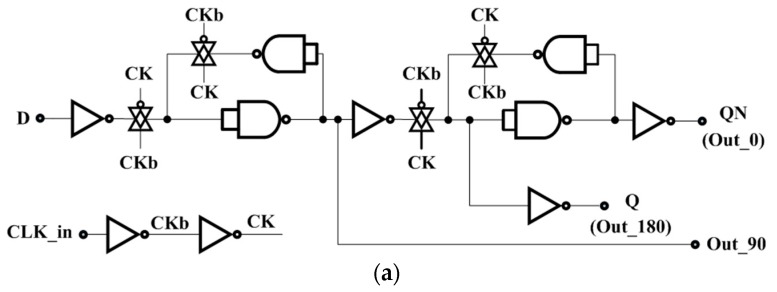

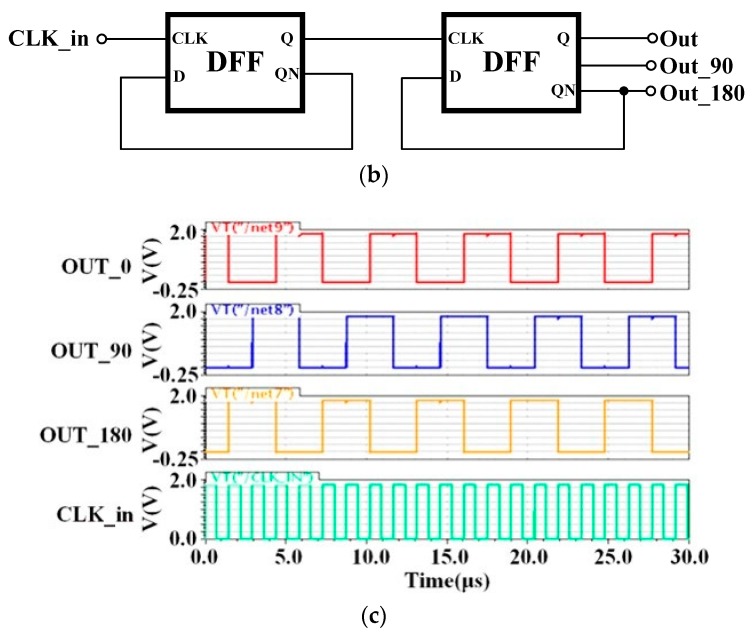
(**a**) D-flip-flop logic diagram; (**b**) Divide-by-four block diagram; (**c**) Divide-by-four simulation results.

The divide-by-four circuit is used to generate the required quadrate signals. The D flip-flop topology is shown in Figure 10a. The nodes “D” and “QN” are connected to form a divide-by-two circuit. The divide-by-four circuit is realized by two divide-by-two circuits in cascade, as shown in Figure 10b. The input clock and output signal waveforms obtained from the simulation of the divide-by-four circuit are shown in Figure 10c. The 0° signal (OUT_0) and the 180° signal (OUT_180) are differential signals that are fed to the LPF, while the 90° signal (OUT_90) is sent to the PFD.

### 3.4. Control Clock Generator

**Figure 11:**
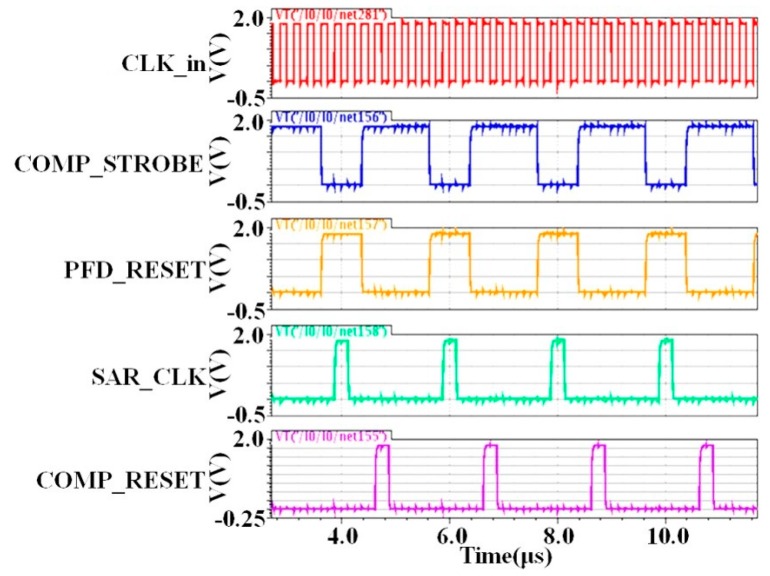
Simulation of the timing control.

The required timing control signals are generated by a control clock generator that is designed with Verilog code and then realized through logic synthesis. The transient waveforms of the input clock and the four timing control clocks, generated from the control clock generator, are shown in Figure 11. The timing control of the ATL is performed as follows. The comparator would be activated by the “Comp strobe”, which is negative-edge triggered. The result of the comparison is then sent to the SAR logic. At the same time, the PFD is deactivated by the “PFD reset”, which is positive-edge triggered. Then, the SAR logic is activated by “SAR CLK” to determine one bit of the binary codes according to the comparison result. Finally, the comparator is deactivated by the “Comp reset”, which is positive-edge triggered.

### 3.5. Automatic Frequency Tuning Loop (ATL)

**Figure 12:**
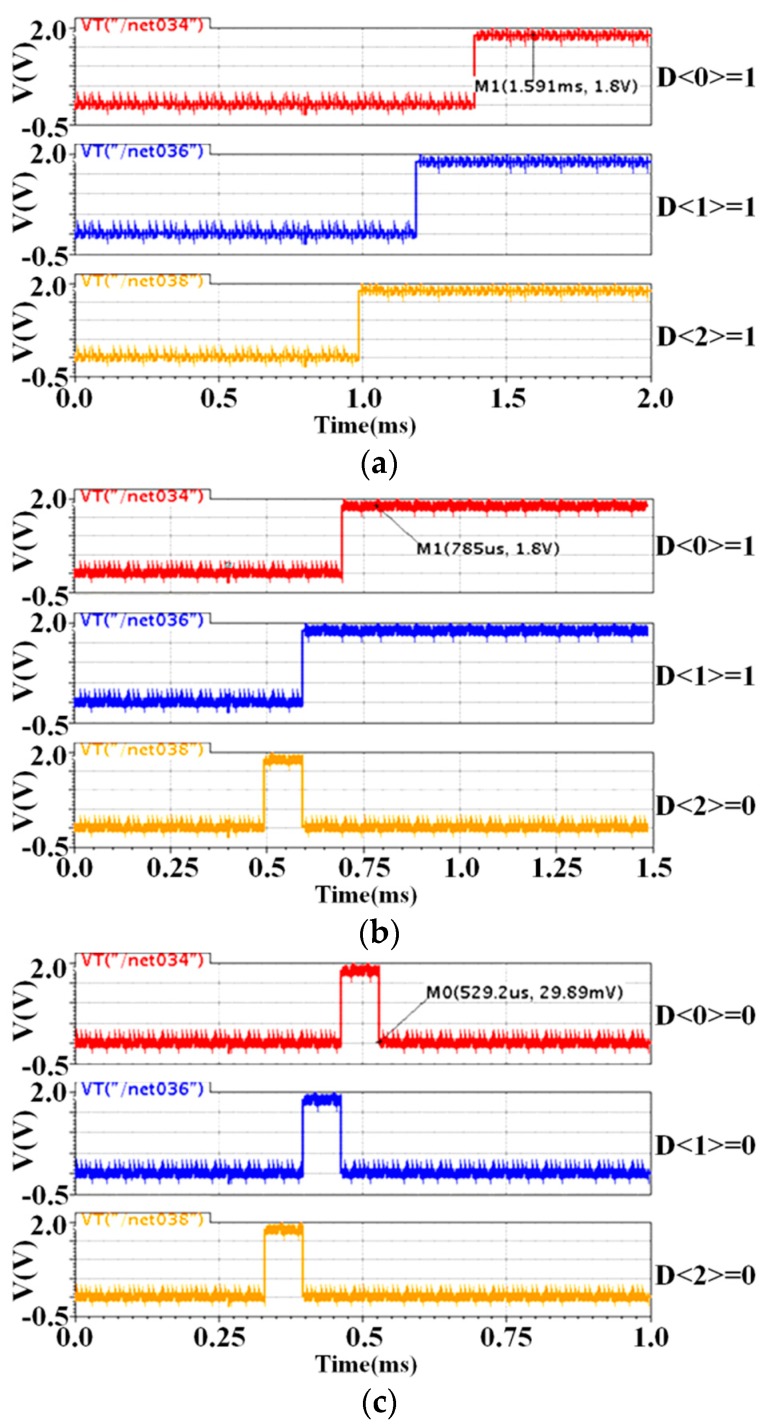
The 3-bit digital code observed in the ATL simulation with an input clock of (**a**) 24.8 kHz, (**b**) 42.8 kHz and (**c**) 60.0 kHz.

When the ATL is applied with different input clocks, the 3-bit digital output of the SAR logic can be observed to verify the frequency tuning function. The digital code is 111 when an input clock of 24.8 kHz is applied to the ATL, as shown in Figure 12a. The digital code is 011 when an input clock of 42.8 kHz is applied to the ATL, as shown in Figure 12b. The digital code is 000 when an input clock of 60 kHz is applied to the ATL, as shown in Figure 12c. Note that the 3-bit digital code controls the variable capacitors in the LPF and determines the corner frequency of the LPF. Therefore, according to the simulation results, the corner frequency of the LPF can be adjusted by the input clock.

## 4. Measurement

### 4.1. Chopper IA

**Figure 13:**
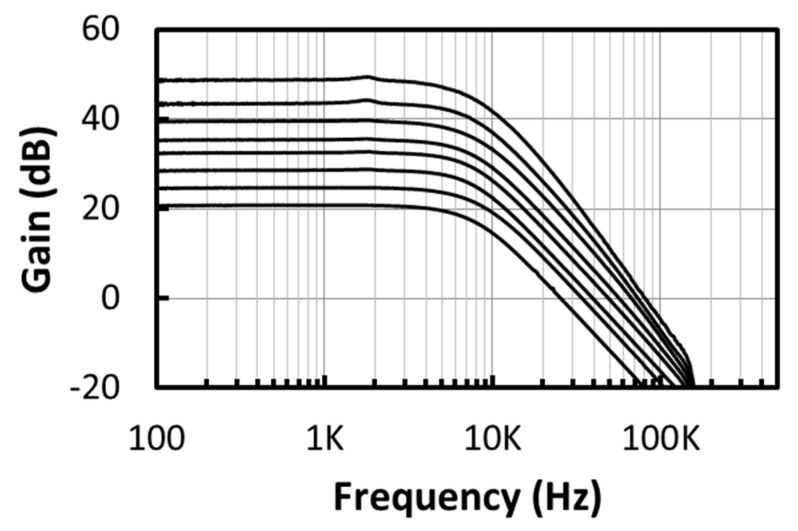
The measured frequency responses of the variable-gain chopper IA.

**Figure 14:**
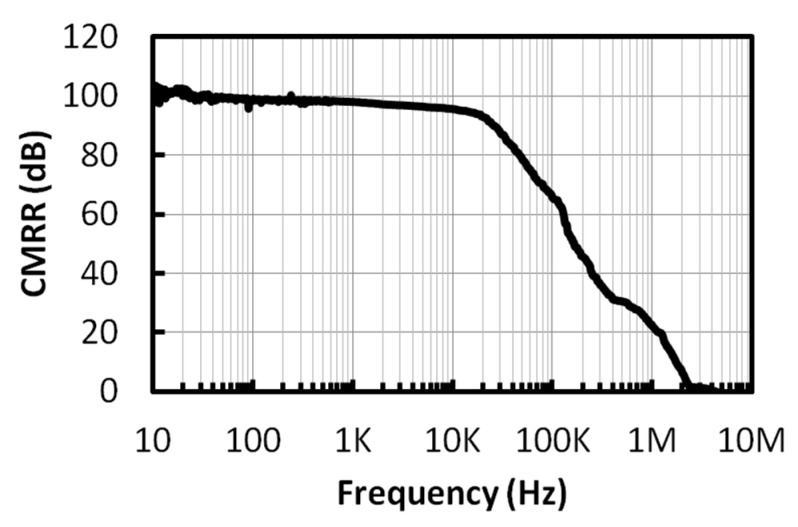
The measured CMRR of the chopper IA.

**Figure 15:**
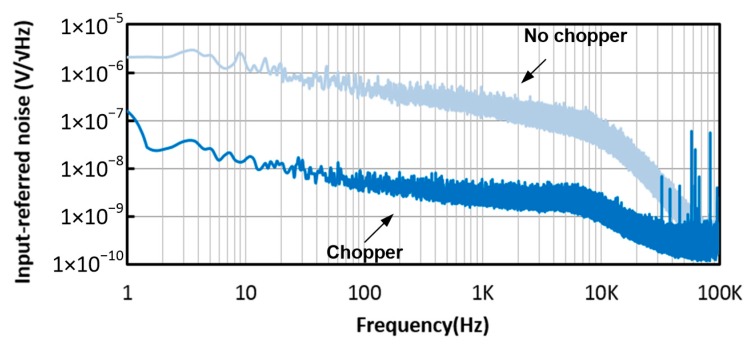
The measured input-referred noise of the chopper IA before and after the chopper modulator is activated.

The measurement of the chopper IA is performed with the Anritsu network analyzer MS4630B. The measured frequency response of the chopper IA is shown in Figure 13, where the gain of the chopper IA can be varied from 20.7 dB to 48.5 dB and the corner frequency is 6.7~7.7 kHz. The measured CMRR of the chopper IA is shown in Figure 14. The CMRR is above 95 dB in the frequency range below 10 kHz. The noise measurement is performed using the R&S UPV audio analyzer (Rohde & Schwarz, Munich, Germany). During the measurement, the ATL is activated to perform the frequency tuning, where an input clock of 40 kHz is provided by the configuration of the 1-MHz on-chip ring oscillator followed by a divide-by-25 circuit, so that the corner frequency of the LPF is set to 10.1 kHz. Three chip samples have been tested. The average of the input-referred noises obtained from these chips, before and after the chopper modulator is activated, is shown in Figure 15. The in-band noise is lowered by 40 dB after the chopper modulator is activated. When the chopper IA operates in the highest-gain mode, the input-referred noise, integrated from 0 to 96 kHz, is 0.28 uVrms.

### 4.2. LPF and ATL

**Figure 16:**
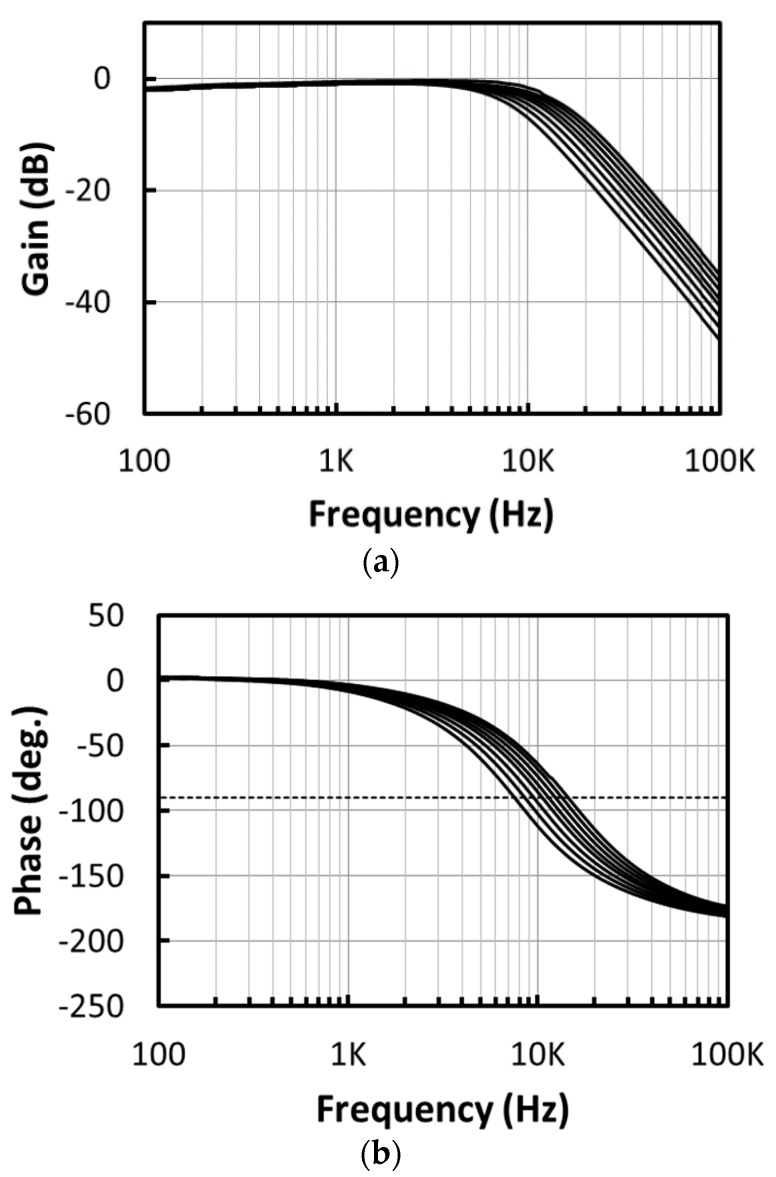
The measured (**a**) gain and (**b**) phase frequency responses of the LPF.

The measurement of the LPF is performed with the Anritsu network analyzer MS4630B (Anritsu Company Inc., Kanagawa Prefecture, Japan). The measured gain and phase frequency responses of the LPF are shown in Figure 16. The corner frequency of the LPF can be varied from 6.7 kHz to 12.8 kHz with a step size of 0.9–1.0 kHz. Note that the frequency at which the phase delay reaches 90° is from 7.5 kHz to 14.4 kHz.

The function of the ATL was verified by observing the digital output of the ATL under different input clocks. During the measurement, the 3-bit digital output of the SAR logic was observed with a logic analyzer, where the input clock was provided by a function generator. The measurement results of digital output under input clocks from 32.8 kHz to 58.4 kHz are listed in Table 1.

**Figure 17:**
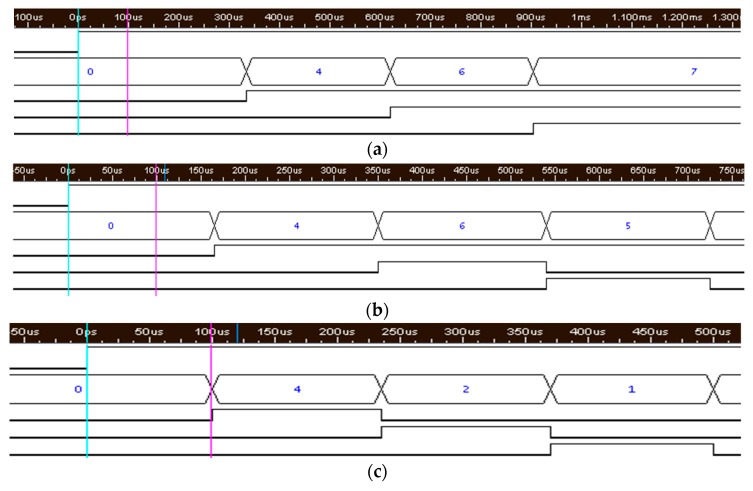
The measurement results of ATL: (**a**) Input clock of 24.8 kHz; ATL digital outputs = 111; (**b**) Input clock of 42.8 kHz; ATL digital outputs = 100; (**c**) Input clock of 60.0 kHz; ATL digital outputs = 000.

**Figure 18:**
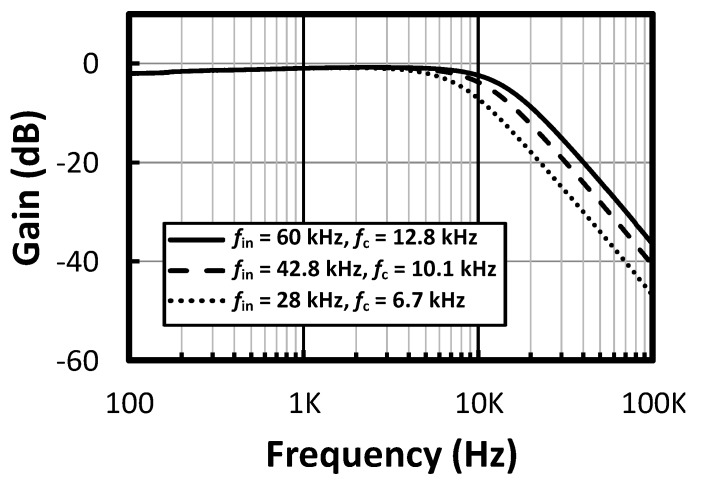
The measured frequency responses of LPF for an ATL input clock of 28, 42.8 and 60 kHz.

The digital outputs of the SAR logic, observed with the logic analyzer under three different clocks, are shown in Figure 17. When an input clock of 24.8 kHz is applied, the observed digital code is 111 after frequency tuning. When an input clock of 42.8 kHz is applied, the observed digital code is 100 after frequency tuning. When an input clock of 60.0 kHz is applied, the observed digital code is 000 after frequency tuning. According to the measurement results, the corner frequency of the LPF can be controlled by the input clock. The frequency response of the LPF for an ATL input clock of 28 kHz, 42.8 kHz and 60 kHz, where the corner frequency of the LPF is adjusted to 6.7 kHz, 10.1 kHz and 12.8 kHz, respectively, is shown in Figure 18.

**Figure 19:**
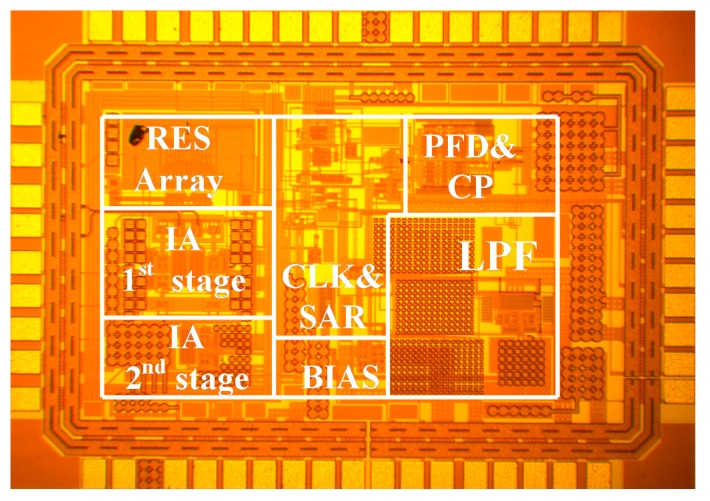
Chip micrograph of the chopper IA with an ATL.

The chopper IA was fabricated with TSMC 0.18 μm CMOS technology. The chip micrograph, where the chip occupies an area of 2.13 mm^2^, is shown in Figure 19. The performance of the chopper IA, along with other previous works for comparison [11,12,13], is summarized in Table 2.

## 5. Conclusions

A low-noise IA is designed and implemented by using the chopper technique, where an LPF is employed to attenuate the up-converted noise at chopping frequency. With an ATL, the corner frequency of the LPF can be controlled with the input clock. The on-chip ring oscillator and dividers are employed to generate the input clock of the ATL and the clock of the chopper modulator, so that the noise reduction can be immune to process variations as the two clocks are correlated. During the measurement, the corner frequency of the LPF is set to 10.1 kHz, while the chopper modulator operates at a clock of 83.3 kHz. The chopper IA consumes 1.1 mW from a supply voltage of 1.2 V. The chopper IA delivers a variable gain from 20.7 dB to 48.5 dB and achieves a minimum bandwidth of 6.7 kHz. The input-referred noise of 0.28 μVrms (0~96 kHz) is achieved.

## Figures and Tables

**Table 1:** Digital Codes under Different Input Clocks.

CLK/4 (kHz)	Digital Codes
>14.6	000
13.7~14.5	001
12.5~13.6	010
11.4~12.4	011
10.4~11.3	100
9.2~10.3	101
8.3~9.1	110
<8.2	111

**Table 2:** Performance Summary of the Chopper IA.

Parameter (Unit)	This Work	[11]	[12]	[13]
Technology (nm)	180	65	180	65
Supply Voltage (V)	1.2	1.2	0.5	1
Chopping Frequency (kHz)	83.3	50	5	5
Gain (dB)	20.7~48.5	34	39.6	100
f3dB (kHz)	6.7~7.7	11	0.25	0.7
Common Mode Rejection Ratio (dB)	>95	>94	>106	134
Power Supply Rejection Ratio (dB)	>95	>100	>73	120
Input-Referred Noise (nV/√Hz)	4.2	37	112	60
Input-Referred Noise (uVrms)	0.205 (0.5–100 Hz) 0.213 (0.5–250 Hz) 0.345 (0.0–96 kHz)	N/A	2.8 (0.5–250 Hz)	6.7 (0.5–100 Hz)
Noise Efficiency Factor [14]	6.13	2.0	8.7	3.9
Power (mW)	1.1	0.0014	0.0013	0.0021
Chip Size(mm^2^)	2.13	N/A	1	0.2
